# BPDCN: When polychemotherapy does not compromise allogeneic CD123 CAR‐T cell cytotoxicity

**DOI:** 10.1002/jha2.149

**Published:** 2020-12-13

**Authors:** Margaux Poussard, Laure Philippe, Maxime Fredon, Elodie Bôle‐Richard, Sabeha Biichle, Florian Renosi, Sophie Perrin, Marie Kroemer, Samuel Limat, Francis Bonnefoy, Etienne Daguindau, Eric Deconinck, Bérengère Gruson, Philippe Saas, Olivier Adotévi, Francine Garnache‐Ottou, Fanny Angelot‐Delettre

**Affiliations:** ^1^ INSERM EFS BFC UMR1098 RIGHT Interactions Greffon‐Hôte Tumeur/Ingénierie Cellulaire et Génique Univ. Bourgogne Franche‐Comté Besançon France; ^2^ Service d'hématologie CH Annecy Genevois Metz‐Tessy France; ^3^ Pharmacy Department,CHRU Besançon University Hospital of Besançon Besançon France; ^4^ Service d'hématologie CHRU Besançon Besançon France; ^5^ Service d'hématologie CHU Amiens Amiens France; ^6^ Service d'oncologie médicale CHRU Besançon Besançon France; ^7^ Laboratoire d'hématologie Etablissement Français du Sang Bourgogne Franche‐Comté Besançon France; ^8^ Laboratoire d'Immuno‐hématologie Etablissement Français du Sang Bourgogne Franche‐Comté Besançon France

**Keywords:** acute leukemia, cell therapy, chemotherapy, immunology, T cells

## Abstract

Blastic plasmacytoid dendritic cell neoplasm (BPDCN) is a rare hematological malignancy with poor prognosis and no treatment consensus. Combining chemotherapy and immunotherapy is a promising strategy to enhance therapeutic effect. Before combining these therapies, the influence of one on the other has to be explored. We set up a model to test the combination of polychemotherapy ‐ named methotrexate, idarubicine, dexamethasone, and L‐asparaginase (MIDA) ‐ and CD123 CAR‐T cell therapy. We showed that CD123 CAR‐T cells exert the same effect on BPDCN models alone, or after MIDA regimen. These data support a preclinical rationale to use immunotherapy after a treatment with polychemotherapy for BPDCN patients.

## 1 INTRODUCTION

Blastic plasmacytoid dendritic cell neoplasm (BPDCN) is an aggressive hematological malignancy derived from plasmacytoid dendritic cell [1]. Median overall survival (OS) is 8‐12 months in the largest patient series in the absence of hematopoietic cell transplantation (HCT) [1, 2]. Clinical presentation is characterized by cutaneous lesions (more than 70% of patients) [3] rapidly progressing to bone marrow and extra‐medullary sites.

No consensus of treatment is established, and even if BPDCN seems to be sensible to standard chemotherapies, relapses occur shortly along with development of resistances. Therapeutic intensification with HCT provides the most prolonged remission [2, 4, 5]. Interestingly, the “anti‐lymphocytic” drugs combination with methotrexate, L‐asparaginase and dexamethasone, already used in NK/T lymphoma, is well tolerated for the majority of patients, can improve OS for up to 30 months, and has the advantage of low toxicity compared to the standard chemotherapies [6, 7, 8]. Moreover, Angelot‐Delettre *et al* have shown significant cytotoxicity of idarubicin *in vitro* in primary BPDCN cells, providing a rationale to combine this drug with NK/T lymphoma regimen to improve the efficacy [9]. Based on these findings, a prospective phase II clinical trial evaluating the combination of methotrexate, idarubicine, dexamethasone, and L‐asparaginase (MIDA) has recently been initiated by a French network on BPDCN (Clinical Trial number: NCT03599960, active recruiting).

The interleukin‐3 receptor alpha chain (CD123) is a promising therapeutic target for BPDCN treatment as it is highly expressed by BPDCN cells, but not at all or weakly by hematopoietic stem cells [10]. Our team recently reported the efficacy and safety of a novel CD28‐41BB‐CD123 CAR‐T (Chimeric Antigen Receptor) cell in relevant BPDCN models [11].

We postulate that CD123 CAR‐T cell therapy could be an alternative to HCT, which due to its aggressiveness and the fact that BPDCN patients are mainly elderly, is not available for the majority of them. However, a frequent adverse event related to CAR‐T cell infusion is the cytokine release syndrome (CRS), whose severity is directly correlated to the tumor burden [12]. So strategies to reduce the tumor burden before CAR‐T cells infusion need to be considered to limit as much as possible the apparition of CRS. One of them could be the use of chemotherapy before CAR‐T cell therapy. However, we first have to show that blasts remain sensitive to CAR‐T cells after being treated by chemotherapy. Thus, we aimed to evaluate the efficacy of CD123 CAR‐T cells on BPDCN cells previously treated with MIDA polychemotherapy.

## 2 MATERIALS AND METHODS

### 2.1 Cell lines

Cell lines were cultured in an appropriate medium (CAL‐1, Dr. Maeda, Nagasaki University, Japan; GEN2.2, Patent N° 0.215.927 and Daudi, ATCC Number: CCL‐213). Primary cells from BPDCN patients were obtained from our French national network (sample collection authorization numbers #DC‐2008‐713 and #DC‐2016‐2791).

### 2.2 CAR construct and T cell transduction

CD123 CAR‐T cell generation was performed as described recently by Bôle‐Richard *et al* [11].

Untransduced T cells (C0) are T lymphocytes from the same donor as CD123 CAR‐T cells, activated and grown under the same conditions. C0 were used as control in all experiments.

### 2.3 Cytotoxicity and functional T cells assays

BPDCN cell lines and primary cells were seeded at 1.10^6^ cells/mL and incubated with or without MIDA's drugs in combination, or as monotherapy for 24 hours (concentrations described in Table S1). Cell viability was assessed by flow cytometry using 7‐AAD and Annexin‐V labeling.

Functional T cells assays were performed against BPDCN cells previously treated or not by MIDA regimen. BPDCN cells were not sorted but a centrifugation was done in order to remove debris and make a number adjustment for further co‐culture with T cells. C0 or CD123 CAR‐T cells labeled by a fixable viability Dye eFluor solution according to manufacturer's protocol (Invitrogen, Carlsbad, CA) were co‐cultured with target cells (BPDCN cells) at the indicated ratio, and cell death was evaluated using 7‐AAD labeling.

T cell‐mediated cytotoxic activity was analyzed by CD107a degranulation assay.

### 2.4 In vivo study

Irradiated NOD/SCID IL2Rgnull‐3/GM/SF (NSG‐S) mice (6‐8 weeks of age, The Jackson Laboratory, Sacramento, CA) were injected with luciferase‐expressing CAL‐1 cell line and treated 2 days later by the MIDA regimen (concentrations described in Table S1). Five days later, mice were infused either with CD123 CAR‐T cells or C0 (intravenous injection). Leukemic progression was monitored weekly by bioluminescence assessment by imaging (IVIS Lumina Series III; Perkin Elmer, Waltham, MA) after luciferin (VivoGlo Luciferin, #P1043, Promega, Fitchburg, WI) intraperitoneal injection. Survival was followed daily. These procedures were carried out in accordance with the guidelines for animal experimentation according to an approved protocol (2019‐001‐SP7PR, Veterinary Service, Ministry for Agriculture, Paris, France).

Further details of materials and methods are available in the supplemental methods.

## 3 RESULTS AND DISCUSSION

**FIGURE 1:**
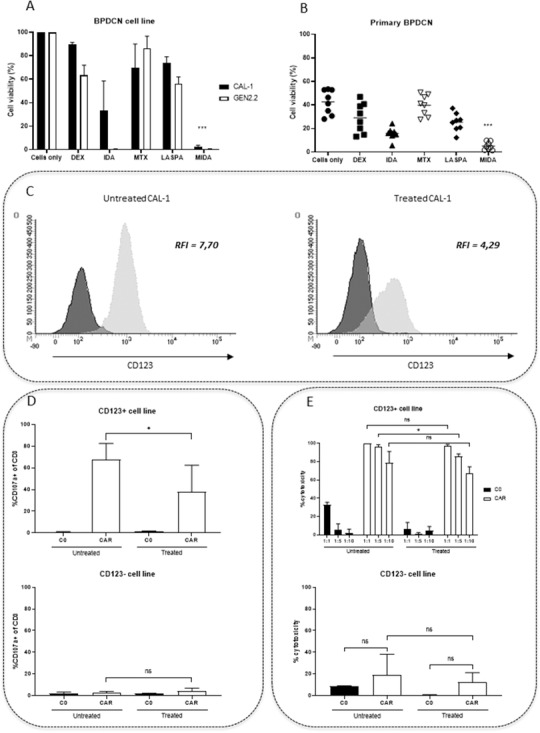
BPDCN cells lines and primary cells exhibit sensitivity to polychemotherapy MIDA regimen and CD123 CAR‐T cells. **A**, Results are expressed as percentage ± SEM of viable cells using AV‐/7‐AAD‐ staining of the CAL‐1 (black) and GEN2.2 (white) cell lines treated with dexamethasone (DEX 0.637 nM), idarubicin (IDA 0.079 μM), methotrexate (MTX 9.9 μM), and L‐asparaginase (LASPA 10UI/mL) as monotherapy or in combination for 24h (MIDA n = 3). Untreated cells were arbitrarily assigned a value of 100% (cells only). **B**, Primary cells of from eight BPDCN patients were treated for 24 hours with chemotherapies alone or in combination in the same conditions as for cell lines. Cell viability was then assessed by flow cytometry (CMF) using AV‐/7‐AAD‐ staining. **C**, CD123 expression level was assessed by CMF on untreated or chemo‐treated CAL‐1. The fluorescence intensity ratio (FIR) was obtained by dividing the mean fluorescence intensity (MFI) of CD123 (light grey curve) by the MFI of the isotype control mAb (dark grey curve). **D**, Untransduced T cells (C0) and CD123 CAR‐T cells (CAR) were cultured with untreated or chemo‐treated cells (CD123^+^ or CD123^neg^) for 6 hours at an E:T ratio of 1:1. The percentage of CD3 cells expressing CD107a after the co‐culture was assessed by CMF. **E**, Untransduced T cells and CD123 CAR‐T cells were co‐cultured with untreated or chemo‐ treated cells (CD123+ or CD123‐) for 24 hours, and the percentage of cytotoxicity was then assessed by CMF Abbreviation: NS, no significant.

MIDA regimen efficacy was assessed *in vitro* on human BPDCN cell lines (CAL‐1 and GEN2.2) and confirmed on primary cells from eight BPDCN patients. MIDA reduces significantly the viability of BPDCN cells compared to untreated cells or chemotherapies used as monotherapy (*P* < *.01 and P* < *.001*, Figure 1A,B). These results demonstrate that MIDA drugs together induce *in vitro* a higher cell death of BPDCN cells. Surprisingly, CD123 expression level is altered by polychemotherapy treatment, with a relative fluorescence intensity of 7.4 ± 0.5 for untreated CAL‐1 cells and 4.5 ± 0.3 for treated ones (Figure 1C). Moreover, the degranulation capacity of CD123 CAR‐T cells is decreased, 67.5% ± 14.9% for CD123 CAR‐T cells co‐cultured with untreated target cells, and 38.0% ± 24.4% for those co‐cultured with treated target cells (Figure 1D).

However, a strong cytotoxicity was equally observed against untreated or treated CAL‐1 regardless of the ratio (1:1 = untreated: 99.8% ± 0.1%; treated: 97.6% ± 1.2%; 1:5 = untreated: 96.0% ± 2.5%; treated: 85.4% ± 2.9%; 1:10 = untreated: 78.6% ± 12.5%; treated: 66.9% ± 7.5%) (Figure 1E). A significant difference can be observed between untreated and treated cells for the 1:5 ratio, however the cytotoxicity remains elevated and above 80%, showing that the cytotoxicity of CD123 CAR‐T cells is not compromised. Thus although the polychemotherapy exposition slightly reduces CD123 expression on target cells, the cytolytic activity of CD123 CAR‐T cells is preserved supporting the high *in vitro* sensitivity of the CAR‐T cells as we previously described [11]. Watanabe *et al* described a similar phenomenon with CAR‐T cells targeting CD20^+^ leukemia and lymphoma cells. They showed that the antigen density required for recognition and lysis by CAR‐T cells was lower than the one required for cytokine production and intracellular signaling of CAR‐T cells [13].

**FIGURE 2:**
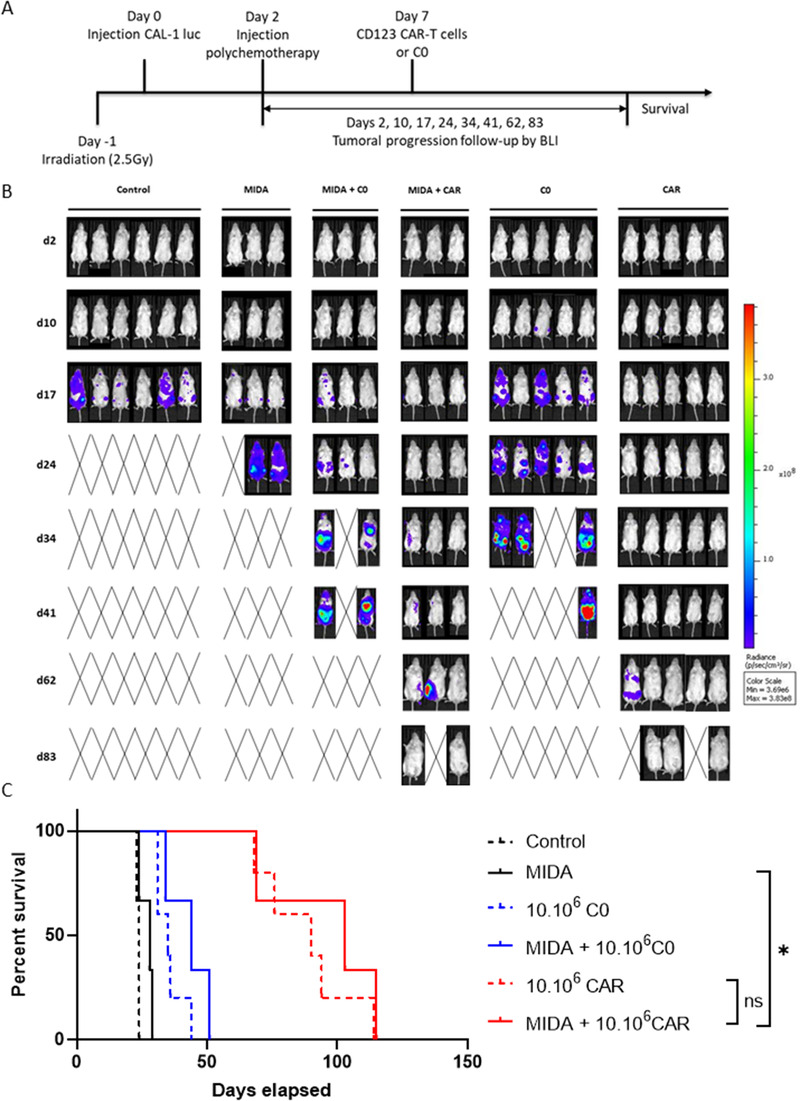
The MIDA protocol does not impair CD123 CAR‐T cells, and their combination improves survival of treated mice. **A**, A diagram showing the different treatment regimens used in (**B and C**). Luciferase+ CAL‐1 cells were injected intravenously into NSG‐S mice. Two days later mice were treated with the MIDA regimen; they received CD123 CAR‐T cells (CD123 CAR‐T) or untransduced T cells (C0) on day 7 at an E:T ratio of 10:1. Groups left untreated or receiving only CD123 CAR‐T cells or untransduced T cells were used as control. Luminescence readings were acquired 2 days following tumor injection and weekly thereafter. **B**, Luminescence of tumor‐ bearing mice from day 2 to day 83. **C**, Kaplan Meier survival curves of mice receiving the indicated treatments. Overall survival of BPDCN inoculated‐mice treated with the MIDA regimen and CD123 CAR‐T cells is shown

To confirm the results obtained in vitro, we performed an in vivo model using NSG‐S mice first treated by MIDA regimen followed or not by CD123 CAR‐T cell or C0 injection (E:T ratio 10:1) (Figure 2A). The groups treated either with CD123 CAR‐T cells alone or after MIDA treatment show a good tumor control, significantly different than the group treated with MIDA alone. Interestingly, no significant difference was observed between the groups treated with the CD123 CAR‐T cells alone and after MIDA regimen (Figure 2B,C). With lower ratio (E:T ratio 1:1), the prior treatment with MIDA seems to potentiate the effect of CAR‐T cells with a better control of tumor progression and a higher survival for the group treated with MIDA+CD123 CAR‐T cells compared to the group treated with CD123 CAR‐T cells alone (Figure S1). As we observed no synergy between MIDA and CAR‐T cells, we could suppose that it came from the molecules composing MIDA regimen. Indeed, idarubicin is known to induce immunogenic cell death which after creating a pro‐inflammatory environment leads in fine to an immunosuppresive one. This will then favor an exhausted profile of CAR‐T cells.

Overall, our *in vitro* and*in vivo* results show that the first line treatment with MIDA does not preclude functionality of CD123 CAR‐T cells against BPDCN cells lines, something that might be expected because of the immunomodulatory properties of methotrexate and dexamethasone [14, 15]. Our data provide a strong rationale to offer CD123 CAR‐T cell therapy to patients that underwent MIDA regimen. MIDA could then be used as a bridge therapy to control and reduce leukemic bulk before CAR‐T cell therapy in order to potentiate CAR‐T cell effects and limit the toxicity of CAR‐T cells by reducing their number administered to the patient (CRS for example).

However, some technical points have to be studied to continue refine this strategy. Our *in vivo* model is an allogeneic one, and experiments in an autologous manner have to be conducted to confirm the allogeneic results. Moreover, the timing of the strategy's setup is calling for other investigations. When should the T lymphocytes be harvested? What is the direct impact of chemotherapy on them and the ability to then generate CAR‐T cells? All these questions are currently investigated by our team.

## CONFLICT OF INTEREST

The authors declare that there is no conflict of interest that could be perceived as prejudicing the impartiality of the research reported.

## AUTHOR CONTRIBUTIONS

Margaux Poussard, Laure Philippe, Maxime Fredon, and Elodie Bôle‐Richard performed the research and designed the research study; Margaux Poussard and Laure Philippe analyzed the data; Francis Bonnefoy assisted with in vivo experiments; Sophie Perrin, Marie Kroemer, and Samuel Limat contributed essential reagents (drugs); Etienne Daguindau, Eric Deconinck, Bérengère Gruson, Francine Garnache‐Ottou, Philippe Saas, and Olivier Adotévi provided guidance and expertise in their respective areas of study; Margaux Poussard, Laure Philippe, and Fanny Angelot‐Delettre wrote the paper; and Francine Garnache‐Ottou, Olivier Adotévi, Etienne Daguindau, Eric Deconinck, and Philippe Saas commented on the paper. Francine Garnache‐Ottou, Olivier Adotévi, and Fanny Angelot‐Delettre supervised the research. All authors provided input, edited, and approved the final version of the paper.

## Supporting information

Supporting Information

**Figure S1 The MIDA protocol does not impair CD123 CAR‐T cells and potentiate their effects on tumor progression. A**, A diagram showing the different treatment regimens used in (**B and C**). Luciferase+ CAL‐1 cells were injected intravenously into NSG‐S mice. Two days later mice were treated with the MIDA protocol; they received CD123 CAR‐T cells (CD123 CAR‐T) or untransduced T cells (C0) on day 6 at a E:T ratio of 1:1. Groups left untreated or receiving only CD123 CAR‐T cells or untransduced T cells were used as control. Luminescence readings were acquired 2 days following tumor injection and weekly thereafter. **B**, Luminescence of tumor‐ bearing mice from day 2 to day 34. **C**, Kaplan Meier survival curves of mice receiving the indicated treatments. Overall survival of BPDCN inoculated‐mice treated with the MIDA protocol and CD123 CAR‐T cells is shown
