# Supplementary material for: BPDCN: When polychemotherapy does not compromise allogeneic CD123 CAR‐T cell cytotoxicity
Source: EJHaem. 2020 Dec 13;2(1):125–30. doi: 10.1002/jha2.149 (PMC9176134; doi:10.1002/jha2.149)
Supplement: Supplementary file 1 — Supporting Information [file JHA2-2-125-s001.docx]

**Supplemental Methods**

***T cell degranulation assay***

CAL-1 and DAUDI cell lines were co-cultured with C0 or CD123 CAR-T cells at the indicated ratio for 6h in presence of Monensin Solution 1X (BioLegend, San Diego, CA, USA), CD107a antibody (Clone H4A3, BD Biosciences, Franklin Lakes, NJ, USA) and Cell Stimulation Cocktail 500X for control conditions (Invitrogen, Carlsbad, CA, USA). Cells were then washed and membrane labeling was performed using CD19, CD3, CD8 antibodies and 7-AAD labeling. Percentage of CD107a positive cells were assessed by FCM.

***Flow cytometry analysis***

All mAbs used for flow cytometry experiments are presented in Supplemental Table 2. The experiments were realized on a LSR Fortessa flow cytometry and analyzed using Diva software (BD Biosciences, Franklin Lakes, NJ, USA). Appropriately matched isotype controls were included for analysis. T cells and CAR-T cells were stained for CD3, CD8 and CD19. BPDCN cells, cell lines and PDX were characterized with CD123 staining. Cell death was evaluated by 7-AAD labeling combined with AnnexinV labeling for some experiments.

***Statistical analysis***

All statistical analyses in this study were performed using GraphPad Prism 7 (GraphPad Software, San Diego, CA, USA). Comparisons between two groups were assessed by Student’s *t* tests. Survival studies were assessed by Kaplan-Meier curves and the log-rank (Mantel-Cox) test. A *P* value <0.05 was considered statistically significant.

***Supplemental Table 1: Concentration of drugs of MIDA regimen.***

Idarubicine, methotrexate and dexamethasone wereobtained from the Pharmacy of the University Hospital of Besançon, France (Dr Kroemer).

L-asparaginase was obtained from Sigma-Adrich, Saint-Louis, Mo, USA.

Concentrations were determined based on literature and adapted to our models.

| **Drugs** | **Concentrations** | |
| --- | --- | --- |
| ***In vitro* experiments** | | |
|  | ***Fig 1A-B*** | ***Fig1 C-E*** |
| Idarubicine | 0.079µM | 0,0079µM |
| Methotrexate | 9.9µM | 0,99µM |
| Dexamethasone | 0.637nM | 0,0637nM |
| L-asparaginase | 10UI/mL | 1UI/mL |
| ***In vivo* experiments** | |  |
| Idarubicine | 1.25.10^-3^mg/mouse |  |
| Methotrexate | 0.065mg/mouse |  |
| Dexamethasone | 0.0625mg/mouse |  |
| L-asparaginase | 12.5UI/mouse |  |

***Supplemental Table 2: Antibodies and reagents used for flow cytometry experiments.***

| **Fluorochrome** | **Target** | **Provider** | **Ref** | **Cells targeted** | **Aim** |
| --- | --- | --- | --- | --- | --- |
| VioBlue | CD3 | Miltenyi Biotec | 130-114-519 | T cells | T cells characterization |
| APC | CD19 | Miltenyi Biotec | 130-113-642 | CAR-T cells |  |
| FITC | CD8 | Sony Biotechnology | 2323520 | T cells |  |
| PE-CY7 | CD123 | Biolegend | 306010 | BPDCN | BPDCN targeting and evaluation of CD123 expression |
| PERCP CY5.5 | 7-AAD | Sony Biotechnology | 2702020 | BPDCN | Cell death evaluation |
| FITC | AnnexinV | Sony Biotechnology | 3804530 | BPDCN |  |
| Annexin V Binding Buffer | / | Sony Biotechnoly | 2711005 | / | / |
