# Supplementary figures and images for: BPDCN: When polychemotherapy does not compromise allogeneic CD123 CAR‐T cell cytotoxicity
Source: EJHaem. 2020 Dec 13;2(1):125–30. doi: 10.1002/jha2.149 (PMC9176134; doi:10.1002/jha2.149)

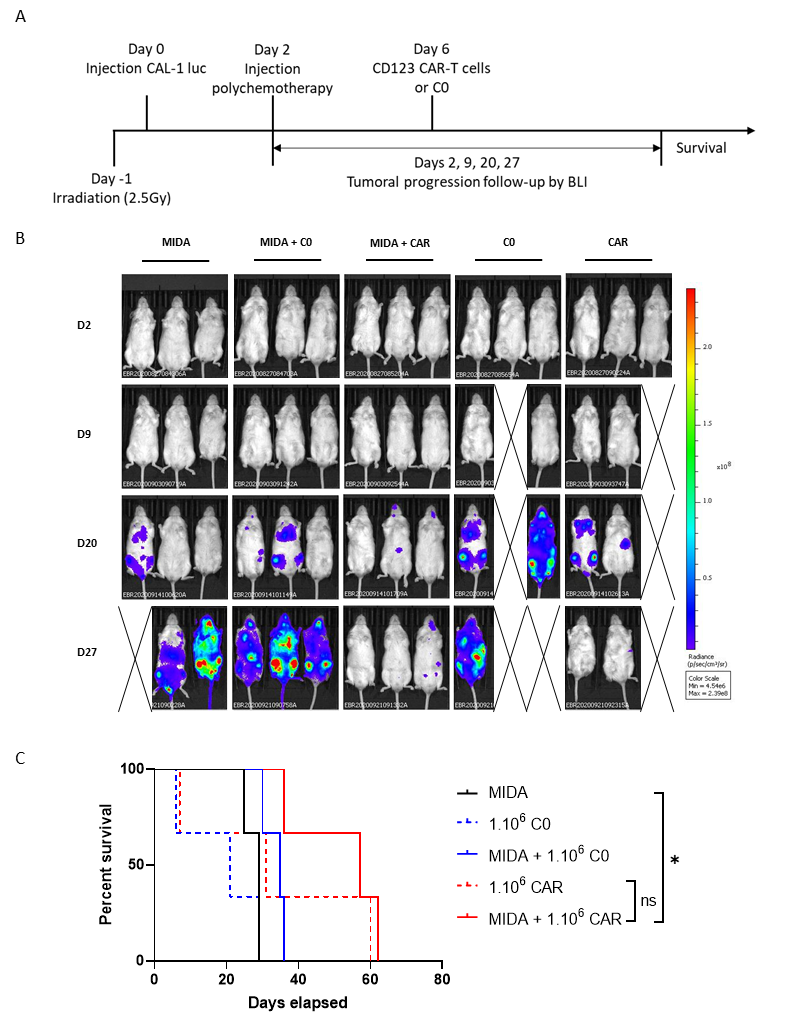

Supplement: Supplementary file 2 — Figure S1 The MIDA protocol does not impair CD123 CAR‐T cells and potentiate their effects on tumor progression. A, A diagram showing the different treatment regimens used in (B and C). Luciferase+ CAL‐1 cells were injected intravenously into NSG‐S mice. Two days later mice were treated with the MIDA protocol; they received CD123 CAR‐T cells (CD123 CAR‐T) or untransduced T cells (C0) on day 6 at a E:T ratio of 1:1. Groups left untreated or receiving only CD123 CAR‐T cells or untransduced T cells were used as control. Luminescence readings were acquired 2 days following tumor injection and weekly thereafter. B, Luminescence of tumor‐ bearing mice from day 2 to day 34. C, Kaplan Meier survival curves of mice receiving the indicated treatments. Overall survival of BPDCN inoculated‐mice treated with the MIDA protocol and CD123 CAR‐T cells is shown [file JHA2-2-125-s002.tif]
